# Electro-Sorption of Hydrogen by Platinum, Palladium and Bimetallic Pt-Pd Nanoelectrode Arrays Synthesized by Pulsed Laser Ablation

**DOI:** 10.3390/mi13060963

**Published:** 2022-06-18

**Authors:** Antonino Scandurra, Maria Censabella, Antonino Gulino, Maria Grazia Grimaldi, Francesco Ruffino

**Affiliations:** 1 Department of Physics and Astronomy “Ettore Majorana”, University of Catania, Via Santa Sofia 64, 95123 Catania, Italy; maria.censabella@ct.infn.it (M.C.); mariagrazia.grimaldi@ct.infn.it (M.G.G.); francesco.ruffino@ct.infn.it (F.R.); 2 Institute for Microelectronics and Microsystems of National Research Council of Italy (CNR-IMM), Via Santa Sofia 64, 95123 Catania, Italy; 3 Research Unit of the University of Catania, National Interuniversity Consortium of Materials Science and Technology (INSTM-UdR of Catania), Viale Andrea Doria 8 and Via S. Sofia 64, 95125 Catania, Italy; agulino@unict.it; 4 Department of Chemical Sciences, University of Catania, Viale Andrea Doria 6, 95123 Catania, Italy

**Keywords:** pulsed laser ablation, nanoparticles, platinum, palladium, bimetallic Pt-Pd, electrochemical hydrogen storage

## Abstract

Sustainable and renewable production of hydrogen by water electrolysers is expected to be one of the most promising methods to satisfy the ever-growing demand for renewable energy production and storage. Hydrogen evolution reaction in alkaline electrolyte is still challenging due to its slow kinetic properties. This study proposes new nanoelectrode arrays for high Faradaic efficiency of the electro-sorption reaction of hydrogen in an alkaline electrolyte. A comparative study of the nanoelectrode arrays, consisting of platinum or palladium or bimetallic nanoparticles (NPs) Pt_80_Pd_20_ (wt.%), obtained by nanosecond pulsed laser ablation in aqueous environment, casted onto graphene paper, is proposed. The effects of thin films of perfluoro-sulfonic ionomer on the material morphology, nanoparticles dispersion, and electrochemical performance have been investigated. The NPs-GP systems have been characterized by field emission scanning electron microscopy, Rutherford backscattering spectroscopy, X-ray diffraction, X-ray photoelectron spectroscopy, cyclic voltammetry, and galvanostatic charge-discharge cycles. Faradaic efficiency up to 86.6% and hydrogen storage capacity up to 6 wt.% have been obtained by the Pt-ionomer and Pd/Pt_80_Pd_20_ systems, respectively.

## 1. Introduction

One of the most important challenges in the development of green energy production and storage is represented by the critical mineral commodities [1]. The list of the strategic minerals for new and emerging technologies could change with time; the most valuable elements include, but are not limited to, lithium, arsenic, gallium, germanium, indium, tellurium, rare-earth elements and cobalt [2]. Those raw materials are strategic for the development of green energy production and storage, but have a high-risk associated with their supply [2]. Moreover, their extraction often produces serious environmental impact and high amount of clean water consumption [3].

Renewable hydrogen production through electro-catalytic water splitting is of paramount importance as sustainable and renewable energy technology can help to overcome the above risks [4]. However, there are currently significant problems in hydrogen-based technology which are represented by the actual technological limits for green production, storage and then distribution. [5,6,7]. Moreover, from an industrial point of view, water splitting in alkaline electrolyte is more appealing, since it overcomes the problem of expensive proton exchange membranes required in acidic electrolytes. Unfortunately, the water splitting in alkaline electrolyte is still challenging due to its slow kinetic properties [8].

Several classes of hybrid nanomaterials have been reported in the literature both for the hydrogen production as well as for its storage at low pressure and ambient temperature [9,10,11]. Compared to the hydrides of metal alloys, the systems based on inorganic electro-catalytic nanomaterials supported onto a carbon matrix are more attractive due to, for instance, their lightweight [12]. Platinum and palladium, particularly in the form of nanoparticles (NPs), are highly efficient electro-catalysts for the hydrogen evolution reaction (HER) by water splitting. Furthermore, they are also hydrogen’s absorbers [13,14,15,16,17,18,19].

Metal NPs prepared by conventional wet chemistry are typically obtained by reduction of a metal precursor by sodium borohydride, or ascorbic acid or ethylene glycol [20]. NPs obtained by chemical reduction may have the surface covered by ligands, surfactants or other unwanted by-products of the reduction reaction. Furthermore, the nanoparticles could be unintentionally doped, incorporating metal ions that limit their electro-catalytic activity. [20]. Conversely, pulsed laser ablation in liquid environment (PLAL) is a versatile and environmentally friendly technique that overcomes the limitation of purity and surface cleanliness present in the NPs produced by conventional wet processes. PLAL is suitable for metal NPs fabrication with a ligand-free surface [21,22,23,24]. In the PLAL method, a laser beam is focused by an optical system on a solid target in a liquid environment, typically water, then the radiation absorbed by the target leads to the formation of an expanding plasma plume, which contains the ablated material and results in a nanoparticle suspension [25]. Moreover, by changing the parameters of the laser (fluence, wavelength, pulse duration) or the liquid media it is possible to obtain nanoparticles with tailored size, physicochemical and morphological properties [25,26,27].

Furthermore, the improvement of the energy efficiency of the electrolysis process in alkaline electrolyte can be obtained by a proper designed ionomer membrane, acting as proton permeable materials or separator [28]. Recently, Hodges and co-workers proposed a porous inter-electrode separator of polyethersulfone 8 μm thick for a capillary-fed electrolysis cell, thus obtaining cost-competitive water splitting in alkaline electrolyte characterized by an energy efficiency of 98% [28].

In this paper we characterized three low-cost systems consisting of novel hybrid nano-electrode arrays of platinum or palladium or Pt-Pd nanoparticles, obtained by PLAL, supported onto graphene paper (GP) [29,30]. Moreover, the effects of a thin proton permeable membrane, enclosing the metal NPs, on the Faradaic efficiency of the electro-sorption reaction of hydrogen in alkaline electrolyte are discussed.

## 2. Materials and Methods

### 2.1. Materials and NPs-GP Preparation

Potassium hydroxide 99.99%, sodium perfluoro-sulfonate ionomer (Nafion™) 5 wt.% solution, and graphene paper 240 μm thick were purchased from Sigma Aldrich Merck (Milan, Italy). NPs suspensions of platinum, palladium and Pt_80_Pd_20_ (wt.%) were prepared by PLAL in water treated in a MilliQ ™ system, characterized by a total organic carbon (TOC) of ≤5 part per billion (ppb) and resistivity of 18.2 MΩ cm. The composition of the Pt_80_Pd_20_ (wt.%) alloy was chosen on the basis of its stability during the laser ablation as sputter target. The detailed methodology, the experimental setup and the conditions used for the NPs preparation were reported in a previous work [30]. NPs-GP nanoelectrode arrays were obtained using pieces of GP of 1 cm × 3 cm. Then, the water-based NPs suspensions were drop casted onto 1 cm^2^ of both sides of GP in hot plate at 100 °C, in air. In details, 29 μg cm^−2^ of platinum, or 5 μg cm^−2^ of palladium, or 5 μg cm^−2^ of Pt_80_Pd_20_ NPs were deposited, obtaining three different sample batches, respectively. The remaining part of the electrode was isolated from the solution by adhesive tape. Another set of nanoelectrode arrays were prepared by drop casting the NPs in water-0.25 wt.% of Nafion suspensions. The latter suspensions were prepared by adding later the stock solution of 5 wt.% Nafion to the water-based NPs suspensions. The estimated average thickness of Nafion film, assuming a density of 1.8 gcm^−3^ for partially hydrated Nafion, is of 0.7 μm [31].

### 2.2. Instrumental Characterization

NPs-GP morphology was investigated by field emission scanning electron microscopy (FE-SEM). A Gemini 152 Carl Zeiss Supra 25 instrument (Jena, Germany) was used. Typically, the analyses were carried out by an acceleration voltage of 5 kV and an aperture size of 30 μm, a working distance of 3 mm, and using an In-lens detector. Transmission Electron Microscopy (TEM) analyses were obtained by a 2010 JEOL Instrument (3-1-2 Musashino, Akishima, Tokyo 196-8558, Japan) employing 200 KeV accelerating voltage. NPs size distribution was determined by the images analysis, using the Gatan Digital Micrograph software version 3.9 (Pleasanton, CA 94588 United States). The mean value of the NPs diameter, 〈D〉, for each sample has been calculated on a statistical population of 900 particles. The associated error consists of the standard deviation on the mean value [30]. 3.5 MV HVEE (High Voltage Engineering Europa, Amersfoort, The Netherlands) Singletron accelerator system was used for the Rutherford Backscattering Spectrometry (RBS) measurements. X-ray diffraction (XRD) measurements were obtained using a Smartlab Rigaku diffractometer (Matsubara-cho, 3-9-12 Akishima-shi, Tokyo, Japan) operating in Bragg–Brentano mode. The X-ray source of Cu Kα radiation with a rotating anode operated at 45 kV and 200 mA.

X-ray photoelectron spectra (XPS) were measured by a PHI 5000 Versa Probe II system ULVAC-PHI, Inc. (2500 Hagisono, Chigasaki, Kanagawa, 253-8522, Japan), and were excited by monochromatized Al Kα X-ray radiation. The photoelectrons were collected at take-off angle of 45° relative to the surface sample holder. The base pressure of the main chamber was 1 × 10^−8^ Pa [32,33]. The instrumental energy resolution was ≤0.5 eV at pass energy of 5.85 eV. The XPS peak intensities were obtained after Shirley background removal. Binding energy scale calibration was achieved by fixing the graphene C 1s main peak at 284.6 eV [32,33]. The atomic concentration analysis was obtained by the peak intensities considering the relevant atomic sensitivity factors [34]. Some X-ray photoelectron spectra were fitted with symmetrical Gaussian envelopes, after subtraction of the background. Data refinement of the fitting process was based on the method of the least squares fitting, carried out until there was the highest possible correlation between the experimental spectrum and the theoretical profile. The residual or agreement factor R, defined by R = [Σ (Fobs − Fcalc)^2^/Σ (Fobs)^2^]^1/2^, after minimization of the function Σ (Fobs − Fcalc)^2^, converged to the value of 0.03 [34].

Electrochemical measurements were performed by Versastat 4 Princeton Applied Research potentiostat (801 South Illinois Avenue Oak Ridge TN, 37830 United States) in air, at 25 °C. Saturated Calomel Electrode (SCE) and platinum electrode were used as reference and counter, respectively. 30 mL of fresh, not de-aerated solution of KOH 1 M was used for each measurement. The electro-catalytic properties of nanostructures towards hydrogen evolution reaction and storage were studied by Cyclic Voltammetry (CV) at scan rate of 20 mVs^−1^ and galvanostatic charge-discharge curved at a current of −100/+100 μA, respectively. The duration of a single charge-discharge cycle was of 600 s.

## 3. Results

### 3.1. Morphology of NPs-GP Nanoelectrode Arrays

**Figure 1:**
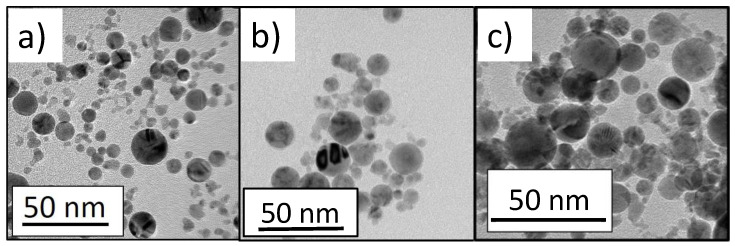
Transmission electron microscopy image of: (**a**) Pt, (**b**) Pd and (**c**) Pt_80_Pd_20_ nanoparticles.

**Figure 2:**
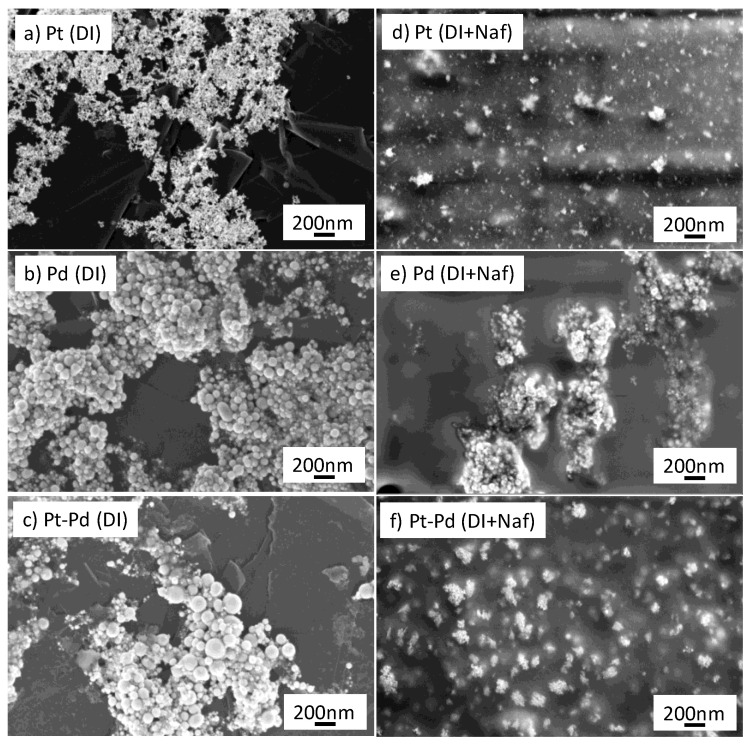
Field emission scanning electron microscopy pictures of NPs-GP nanocomposites: (**a**–**d**) Pt in deionized water and in Nafion solution; (**b**–**e**) Pd in deionized water and in Nafion solution; (**c**–**f**) Pt_80_Pd_20_ in deionized water and in Nafion solution.

Figure 1a–c report the TEM images of the Pt, Pd and Pt_80_Pd_20_ NPs, respectively. The shape of the NPs is almost spherical and the average size is 10 ± 2 nm for Pt, 12 ± 2 nm for palladium and 11 ± 5 nm for Pt_80_Pd_20_, respectively. More detailed analysis of the NPs has been reported in a previous work by the authors [30]. Figure 2a–c report the morphology, studied by FE-SEM, of the NPs-GP composite systems obtained by water-based suspensions of Pt, Pd and Pt_80_Pd_20_ NPs, respectively. It is worthy of note that the FE-SEM pictures of Figure 2a-c show the presence of some larger NPs than those shown in the TEM pictures. The reason of this apparent discrepancy is due to the tail in the size distributions presented by the PLAL synthesized nanoparticles [27,30].

Figure 2d–f show the FE-SEM morphology of the NPs-GP composite systems obtained by casting NPs suspension in water containing 0.25% wt. of Nafion. Clearly, the Nafion contribute significant to the dispersion of NPs on the surface. The dispersion is produced by the negatively charged sulphonic groups of Nafion which are present on the surface of the coated NPs and, therefore, repel them from each other and avoid their aggregation. This result is of relevant importance in the electrochemical behavior of the PNs-GP towards hydrogen production and storage (vide later on).

**Figure 3:**
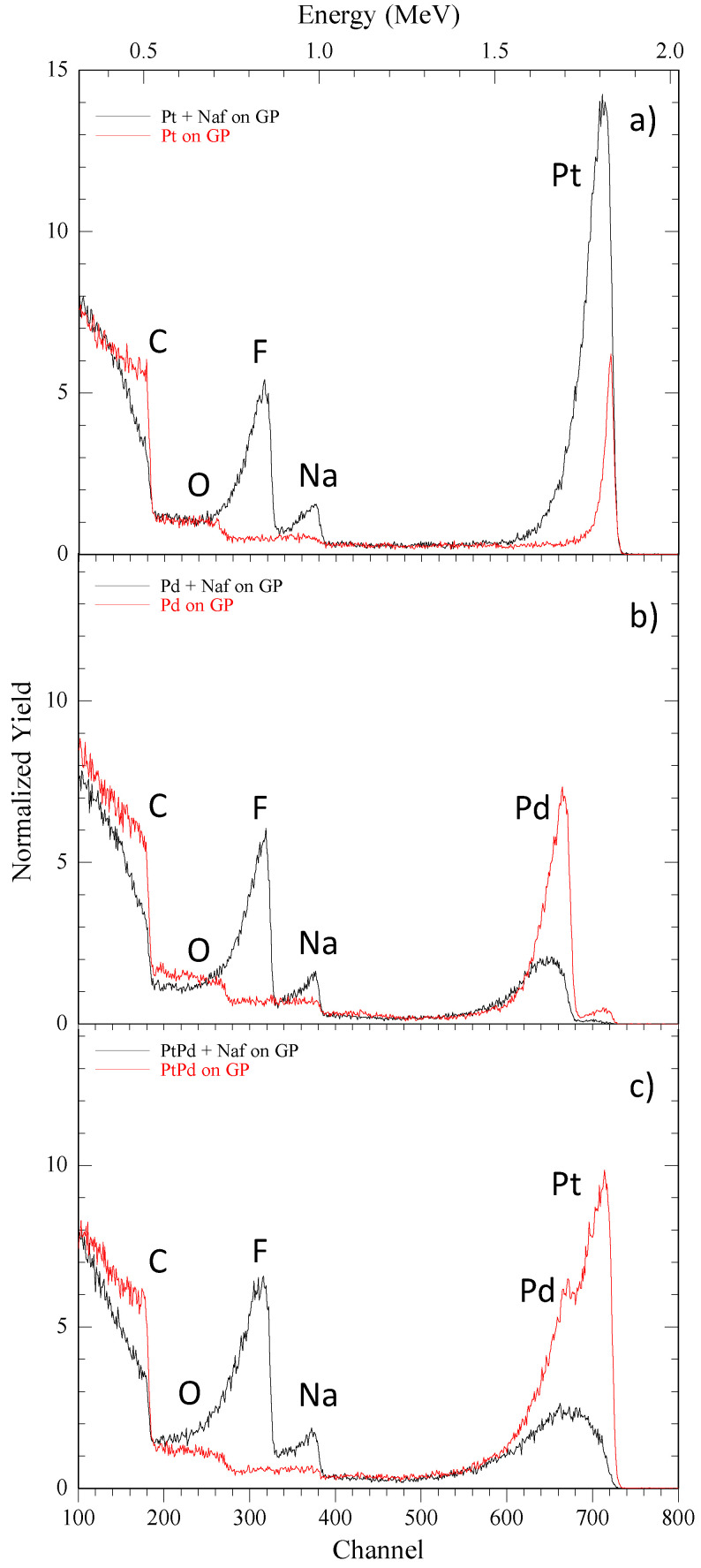
Rutherford backscattering spectra of NPs-GP composite systems obtained by water (red lines) and water-0.25% wt. Nafion suspensions (black lines): (**a**) Pt; (**b**) Pd; (**c**) Pt_80_Pd_20_.

Figure 3a–c shows the RBS spectra of the NPs-GP systems. RBS spectra were simulated by using XRump software, [35] which furnished the composition and identified the element present on the outermost layer of surface. The spectra show signals of C, O, S, Na, Pd, and Pt, as marked in the following Figure (simulation not shown). The spectra of the NPs-GP systems obtained by water suspension show a weak signal of sodium, whose origin may be attributed to the process of GP fabrication. Furthermore, the metal nanoparticles produce sharp peaks of the backscattered He^+^ ions thus confirming the homogeneity and contiguity of the NPs arrays. In contrast, the presence of Nafion produces a significant broadening of the peaks associated to the metal nanoparticles, with a tail on the lower side of the energy scale. The low energy tail in the spectra (below 1.6 MeV) is attributed to metal NPs enclosed in the Nafion film [36].

### 3.2. Structure of PLAL NPs

**Figure 4:**
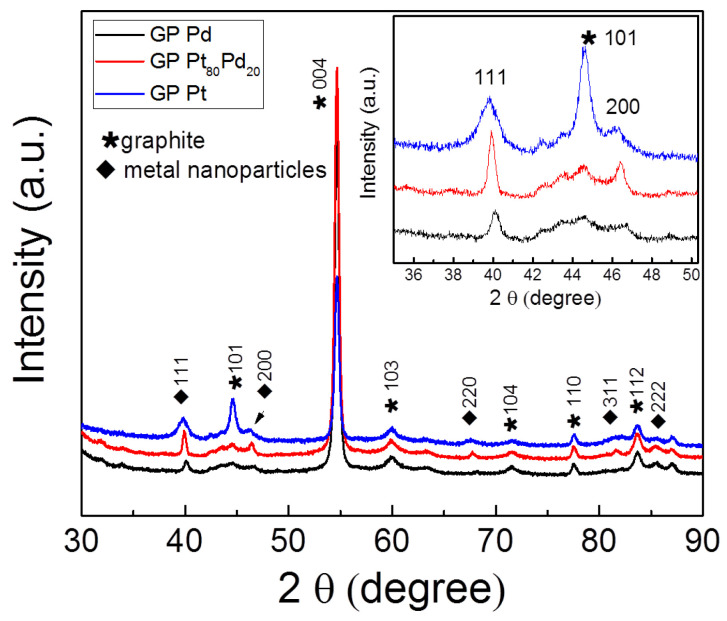
XRD patterns of platinum, palladium, Pt_80_Pd_20_ nanoparticles supported onto graphene paper.

Figure 4 shows the XRD patterns of the platinum, palladium and Pt_80_Pd_20_ NPs-GP composite systems. The XRD patterns show the signal at 2θ with the values of 44.52, 54.66, 59.85, 71.47, 77.40, 83.53, corresponding to 101, 004, 103, 104, 110, 112 reflections which are assigned to the graphitic phase of the GP [37]. The patterns in Figure 4 do not contain specific features to be attributed to single graphene layers or graphene oxides. Additional peaks (marked by lozenge in Figure) are attributed to the 111, 200, 220, 311 and 222 reflections of the metal nanoparticles [38,39].

The inset shows the enlarged region containing the most intense 111 and 200 peaks of metal nanoparticles. The 111 and 200 reflection peaks were found at 39.82°, 46.08° for the platinum, and at 40.13°, 46.62° for palladium, and match those of Face-Centered Cubic (FCC) structure of platinum (JCPDF 04-0802) and of palladium (JCPDF 46-1043), respectively. The Pt_80_Pd_20_ NPs show the 111 and 200 reflection peaks at 39.94° and 46.43°, respectively. According to the composition of the intermetallic nanoparticles, the latter values are included between those of the two pure metals. The XRD results show the crystalline nature of all of the NPs considered here. In particular, the width and position of the peak 111 reflect the different composition and average size of the metallic NPs [38]. Furthermore, the present bimetallic NPs structure does not show the core-shell type, as described elsewhere [30].

### 3.3. Surface and Electronic Structure of NPs

The surface of the Pd-GP, Pt-GP and Pt_80_Pd_20_-GP have been investigated by XPS, which provides information on the electronic structure and allows estimation of the surface elemental composition, once the relevant atomic sensitivity factors have been taken into account [32,33,40,41].

**Figure 5:**
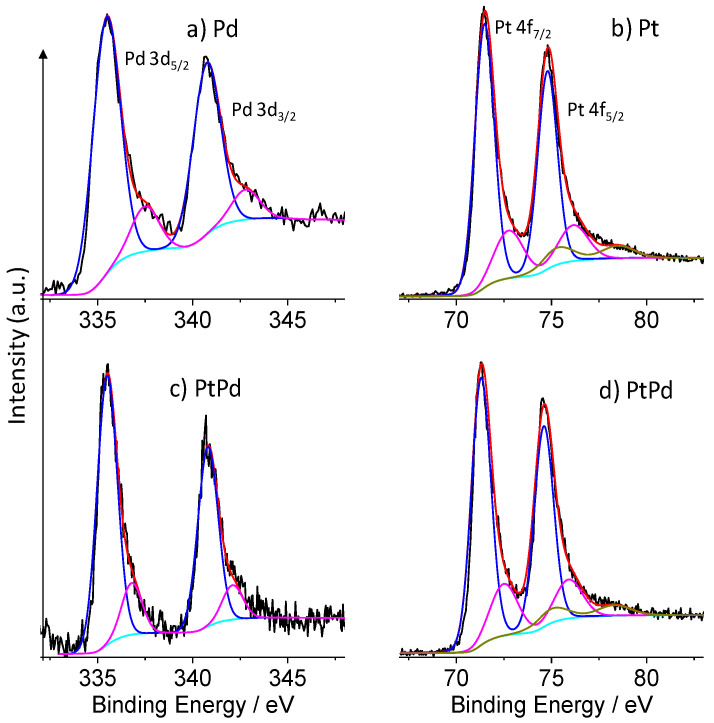
Al-Kα excited photoelectron spectra of Pd 3d binding energy region of: (**a**) Pd-GP and (**c**) Pt_80_Pd_20_-GP. The 3d_5/2_—3d_3/2_ spin-orbit doublets (blue and magenta line) refer to the Pd^0^ and Pd(II) states, respectively; 4f binding energy region of: (**b**) Pt-GP and (**d**) Pt_80_Pd_20_-GP. The 4f_7/2_—4f_5/2_ spin-orbit doublets (blue, magenta and dark yellow line) refer to Pt^0^, Pt(II) and Pt(IV) states, respectively. The cyan line refers to the background and the red line superimposed to the experimental black profile refers to the sum of all of the Gaussian components.

Figure 5a shows the XPS of the Pd-GP in the Pd 3d binding energy region. The spectrum was deconvoluted with the superposition of two doublet components; a dominating doublet at 335.5 and 340.8 eV (5.3 eV spin-orbit coupling) is associated to the 3d_5/2,3/2_ of metallic palladium (Pd^0^) states and a doublet at 337.5 and 342.8 eV (5.3 eV spin-orbit coupling) is associated to Pd(II) due to a partial surface oxidation of palladium [42,43].

Figure 5b shows the XPS of the Pt-GP in the Pt 4f binding energy region. The XPS spectra of Pt 4f were deconvoluted using three doublet components. The main doublet at 71.3 and 74.6 eV (3.3 eV spin-orbit coupling) is assigned to the 4f_7/2,5/2_ of zero valent Pt^0^ [44], while the doublet at 72.6 and 76.0 eV (3.4 eV spin-orbit coupling) is attributed to Pt(II) species [44,45]. The higher doublet at 75.2 and 78.5 eV (3.3 eV spin-orbit coupling) is assigned to Pt(IV) species [45].

Figure 5c shows the XPS of the Pt_80_Pd_20_-GP in the Pd 3d binding energy region. The Pd 3d level was deconvoluted with the superposition of two doublet components: a dominating doublet at 335.5 and 340.8 eV (5.3 eV spin-orbit coupling) is associated to the metallic palladium (Pd^0^) states, and the doublet at 336.8 and 342.1 eV (5.3 eV spin-orbit coupling) is associated to Pd(II) due to a partial surface oxidation of palladium [42,43]. Gaussian relative intensities are almost coincident with those observed for the Pd-graphene sample, but the higher binding energy doublet (336.8–342.1 eV) is at 0.7 lower binding energy values with respect to that observed for the Pd-graphene-sample. This observation agrees with the somewhat larger electronegativity of palladium (1.40) with respect to that of platinum (1.35) and highlights the establishment of a chemical bond between the two metals that results in electron donation from platinum to palladium.

Figure 5d shows the XPS of the Pt_80_Pd_20_-GP sample in the Pt 4f binding energy region. The XPS spectrum of Pt 4f was deconvoluted using three doublet components. The main doublet at 71.3 and 74.6 eV (3.3 eV spin-orbit coupling) is assigned to the zero valent Pt^0^ [44], while the doublet at 72.5 and 75.9 eV (3.4 eV spin-orbit coupling) is attributed to Pt(II) species [44,45]. The higher doublet at 75.0 and 78.3 eV (3.3 eV spin-orbit coupling) is assigned to Pt(IV) species [45]. Both B.E. values and Gaussian relative intensities are similar to those observed for the Pt-GP sample. Table 1 summarizes the component position used in the spectra deconvolution.

**Figure 6:**
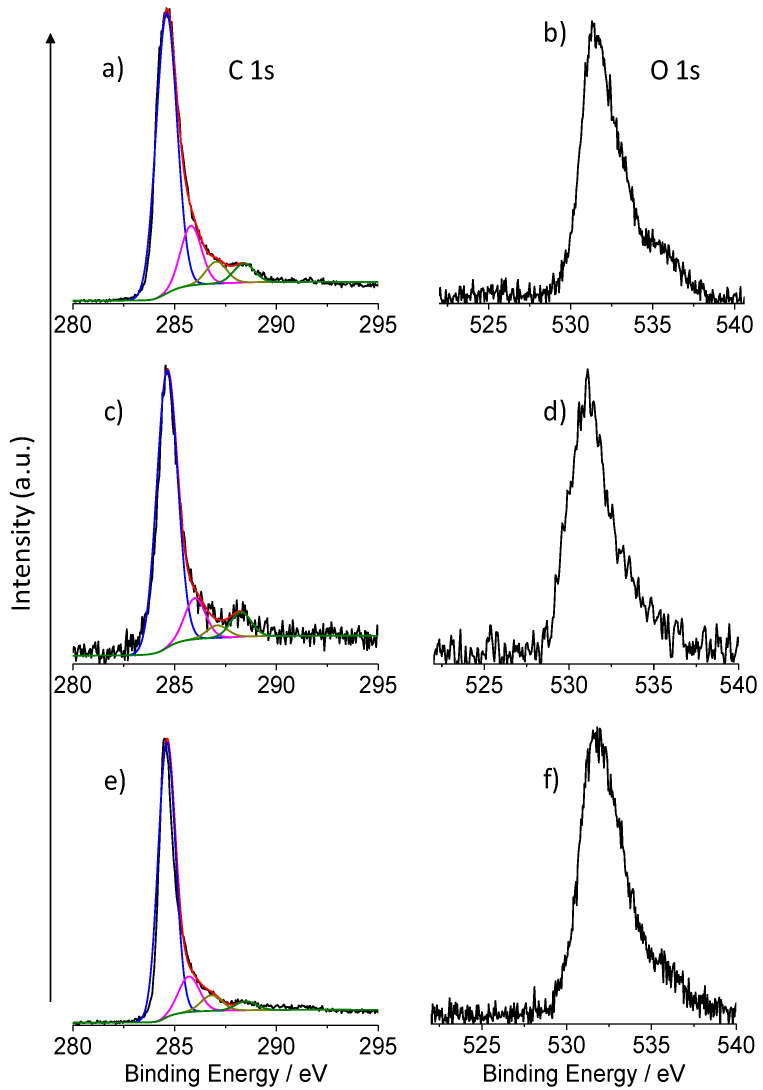
Al-Kα excited XPS in the binding energy region of C 1s: (**a**) Pd-GP; (**c**) Pt-GP; (**e**) Pt_80_Pd_20_-GP. The component positions are reported in Table 1. O 1s binding energy region of: **(b**) Pd-GP; (**d**) Pt-GP; (**f**) Pt_80_Pd_20_-GP.

Figure 6a,b show the high-resolution XPS of the Pd-GP in the C 1s and O 1s binding energy (B.E.) regions, respectively. A careful fitting of the experimental profile of the C 1s signal required four Gaussian components centered at 284.6, 285.8, 287.1 and 288.4 eV, respectively (Figure 6a and Table 1). The first component (284.6 eV) is due to *sp^2^* carbon states [45,46]. The other peaks at 285.8, 287.1 and 288.4 eV are assigned to the C-OH, C=O and O-C=O functional groups present on the surface of the graphene paper [46,47].

The XPS peak in the O 1s core level binding energy (Figure 6b) is centered at 531.5 eV and shows some high energy broadening that clearly points to the presence of more components due to O-C=O and -OH groups of the graphene surface, to the oxygen-bonded to palladium (Pd-O) and to the overlapping Pd 3p_3/2_ spin-orbit component of the Pd metal [48,49]. The additional high binding energy shoulder at 535.5 eV is attributed to the presence of some H_2_O molecules on the sample surface [32].

Figure 6c shows the high-resolution XPS of the Pt-GP in the C 1s energy region. An accurate fitting of the spectrum revealed the presence of four components at 284.6, 286.0, 287.1 and 288.3 eV, respectively. These components are at B.E. values similar to those observed for the Pd-graphene sample and due to the same electronic states. The main difference with respect to the previous related XPS results is due to a decreased relative intensity of the component at 287.1 eV (due to the C=O graphene substituent).

The main O 1s signal for the Pd-GP (Figure 6d) lies at 531.1 and is due to the O-C=O groups of the graphene surface and to the oxygen-bonded Pt (Pt-O). The additional high binding energy shoulder is attributed to -OH groups and some water molecules on the graphene surface [48].

Figure 6e shows the high-resolution XPS of the Pt_80_Pd_20_-GP in the C 1s energy region. An accurate fitting of the spectrum profile revealed the presence of four components at 284.6, 285.7, 286.9 and 288.5 eV, respectively. These components are at B.E. values similar to those observed for the Pd-graphene and Pt-graphene samples and due to the same electronic states. The main difference with respect to the previous related XPS results is due to a decreased relative intensity of the last component (288.5 eV, due to the O-C=O graphene substituent). This datum is likewise in agreement with the lower oxygen atomic concentration (Table 2).

Figure 6f shows the O 1s spectrum for the Pt_80_Pd_20_-GP sample. The O 1s core level centred at 531.8 eV shows some broadening that clearly points to the presence of more components due to O-C=O and -OH groups of the graphene surface, to the oxygen-bonded to platinum (Pt-O) and to palladium (Pd-O) and to the overlapping Pd 3p_3/2_ spin-orbit component of the Pd metal. The additional high binding energy shoulder at 534.0 eV is attributed to the presence of some H_2_O molecules on the sample surface [32]. Table 2 reports the surface composition obtained by the XPS analyses. Notable, the platinum content in the Pt-GP is higher than that of palladium and platinum in the respective Pd-GP and Pt_80_Pd_20_-GP systems. The data reflect the nominal amount of NPs casted onto the surface of GP (vide experimental section). Moreover, the surface composition of the bimetallic NPs that has the bulk composition of Pt_68.3_Pd_31.7_ by atomic%, is slightly enriched in palladium with respect to the bulk composition, indicating the presence of the segregation of the latter metal.

### 3.4. Electro-Sorption and Galvanostatic Charge-Discharge Properties

**Figure 7:**
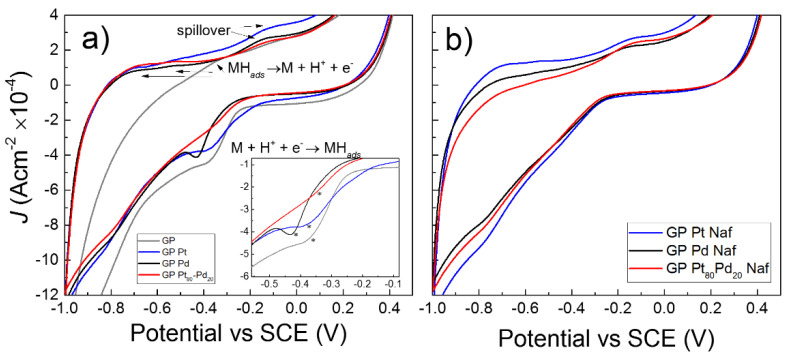
(**a**) Cyclic voltammograms of GP and NPs-GP; (**b**) cyclic voltammograms of NPs-GP obtained by suspension in water 025% wt. Nafion. Conditions: KOH 1 M; scan rate 20 mVs*^−^*^1^. The inset shows the peak position of hydrogen ion adsorption and reduction, marked by asterisk.

The hydrogen electro-sorption reaction by the NPs-GP electrodes was performed in alkaline electrolyte. In alkaline electrolyte the reaction is less efficient than that in acidic electrolyte, as consequence of the slow step of reaction consisting in the water dissociation into OH^-^ and H^+^ (Equation (1)) [50]. However, from an industrial point of view, alkaline electrolytes are more interesting since they overcame the problem of expensive proton exchange membranes required in acidic electrolyte [50]. Figure 7a shows the cyclic voltammograms recorded in the potential region comprises between −1 and 0.4 V by the NPs-GP nanoelectrode arrays based on platinum, palladium or Pt_80_Pd_20_. For comparison, the voltammogram obtained by the GP alone was included in the Figure. In alkaline electrolyte hydrogen ions are adsorbed and reduced onto the metal surface according to the Volmer-Heyrovsky mechanism summarized by Equations (1) and (2), where M represents the metals or Pt_80_Pd_20_ alloy [51].

H_2_O ⇆ H^+^ + OH^−^ (1)

M + H^+^ + e^−^ ⇆ MH_ads_ (2)

The peaks associated with the adsorption and reduction of the H^+^ ions were found at potentials vs. SCE of −0.38 V (GP alone), −0.37 V (Pt), −0.43 V (Pd), and −0.35 V (Pt_80_Pd_20_), respectively. The inset in Figure reports enlarged portion of voltammograms containing the peak position marked by asterisk. The peak of H^+^ adsorption and reduction by the bimetallic alloy has the lowest potential value, showing a greater efficiency of this system with respect to the others in the catalysis of this process. According to the Butler-Volmer Equation, the shift of the cathodic peak towards higher potential values is accompanied by an increase of its full width at half maximum (FWHM). Thus, the peak of hydrogen reduction in the voltammogram by bimetallic system is characterized by larger width, compared to the peaks obtained by the platinum or palladium single catalysts [52]. The peaks of hydrogen desorption and oxidation to H^+^ ion are located at potentials of −0.31 V (GP alone), −0.68 and −0.49 V (Pt), −0.68 and −0.53 V (Pd), −0.64, and 0.53V (Pt_80_Pd_20_), respectively. Conversely to what observed in the voltammogram of GP alone, the voltammograms of the NPs-GP electrodes show two anodic peaks that are attributable to the mechanism involving the desorption of MH_ads_ and oxidation of the H· radical to the H^+^ ion on different crystallographic planes of the NPs surface [53]. Notably, additional peak at about −0.13 V for all systems containing NPs is visible in the voltammograms. This peak is attributable to a significant process of hydrogen spillover present in the investigate systems [54]. With the spillover mechanism, the hydrogen passes from the NPs to the GP, which represents the main absorber for storage (Equation (3)).

MH_ads_ + GP ⇆ M + GPH_ads_ (3)

Notable, due to the nature of hybrid nanoelectrode array of these systems, the peaks present in the voltammograms are not well pronounced [55]. This is further evident in voltammograms of the systems obtained using Nafion (Figure 7b), that are characterized by finely dispersion of the metal NPs.

**Figure 8:**
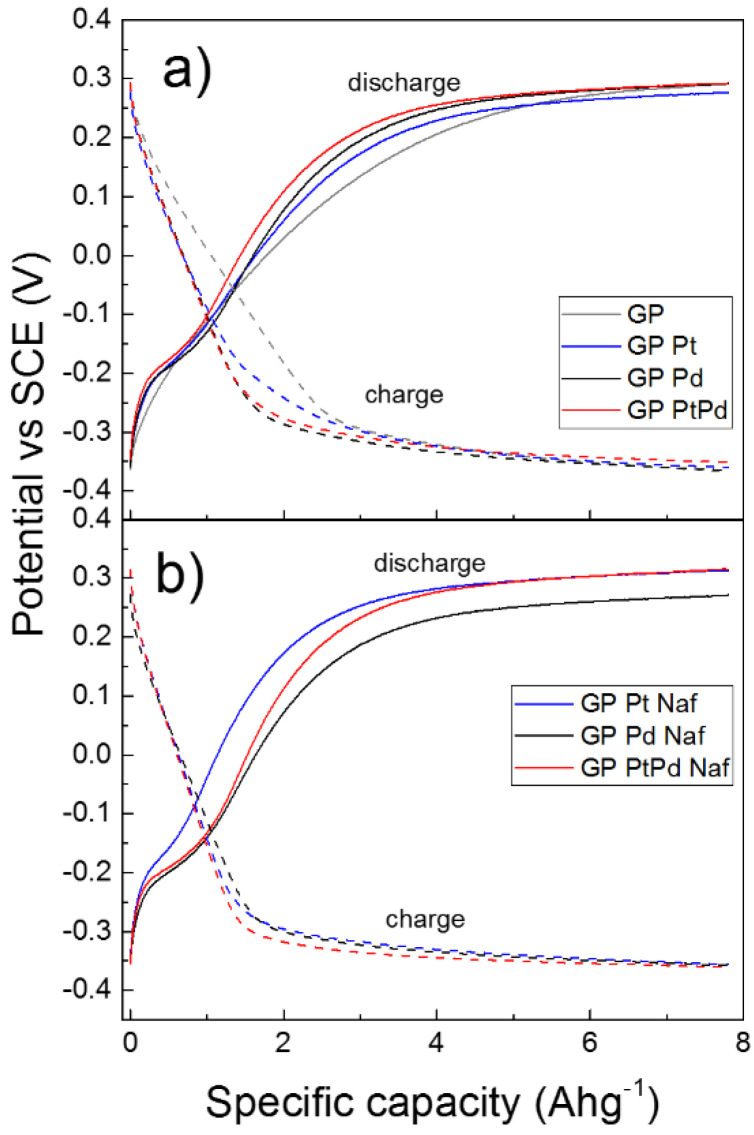
(**a**) galvanostatic charge and discharge curves of GP and NPs-GP; (**b**) galvanostatic charge and discharge of NPs-GP obtained by suspension in water 025% wt. Nafion. Conditions: KOH 1 M; current 100 μA. The specific capacity was calculated with respect to the mass of 1 cm^2^ of GP.

Further investigation of the electrochemical properties of the systems was carried out by galvanostatic charge and discharge measurements. Figure 8a shows the galvanostatic charge and discharge curves obtained by the various NPs-GP systems, in comparison to the GP alone. The Figure reports the electrode potential as function of the specific capacity. In this experiment, the electrodes were charged at a capacity up to 7.8 Ahg*^−^*^1^ and then discharged. We reported the fifth cycle of charge-discharge processes when the stabilization of the electrode potential occurs. The specific capacity was calculated on the basis of Faraday’s law, taking into account the mass of 1 cm^2^ of graphene paper [56,57,58]. At this value of specific capacity, the condition of potential stabilization is reached both in the charge as well as in the discharge curves. From the curves of Figure 8a it can be seen that at the initial stage of both charge and discharge processes (e.g., roughly for the first 4 Ahg*^−^*^1^), the Pt-GP system assumes potentials greater than that of the GP alone, showing higher kinetics (as expected for the presence of the catalysis offered by the platinum nanoparticles). The Pd-GP systems and, in particular, Pt_8_Pd_20_-GP are further efficient with respect to the charging and discharging processes even with respect to the Pt-GP. This is particularly evident if we take into account the different amount of NPs casted per square centimeter of GP (vide experimental section). In particular, assuming that the charge is entirely used for the reduction reaction of H^+^, in agreement to that assumed in previous works, [56,57,58] it is possible to obtain that the percentage of hydrogen developed at the steady state potential of polarization curves, accumulated or transferred to the GP, is equal to 1 wt.% for the platinum, and 6 wt.% both for the palladium and the bimetallic Pt_80_Pd_20_ (vide later on Table 3). It is worthy of notice that, since the GP used in this study has a thickness of 240 μm, it is likely that it mainly acts as a conductor of the electrical charges, while only a very thin layer close to the electrical double layer is involved in the hydrogen absorption process [59].

Figure 8b shows the galvanostatic charge and discharge curves obtained by the NPs-GP systems casted by water-0.25 wt.%. Nafion suspension. In these systems the thin layer of Nafion surrounding the NPs surface acts as proton exchange membranes (Equation (4)). In particular, Nafion is H^+^ conductive and then increases the activity of hydrogen ions neighbor of the NPs surface (Equation (5)):

(CF_n_O_m_)-SO_3_^−^ + H_2_O ⇆ (CF_n_O_m_)-SO_3_H + OH^−^ (4)

(CF_n_O_m_)-SO_3_H + M + e^−^ ⇆ MH_ads_ + (CF_n_O_m_)-SO_3_^−^ (5)

where (CF_n_O_m_)-SO_3_^-^ represents the ionomer in the anionic form and (CF_n_O_m_)-SO_3_H represents the ionomer interacted with the H^+^. Table 3 reports the electrochemical performances of our systems derived from the curves of Figure 8a,b. In detail, the galvanostatic charge and discharge capacities of the various NPs-GP systems are compared. For the charging capacity calculation, we assumed the start of the charging process when the electrode potential reaches a value of −0.3 V vs. SCE. This value corresponds roughly to the 97% of the steady state potential. In the discharge process the cut-off potential was assumed at 0.25 V vs. SCE that corresponds to 90% of the steady state potential. Faradaic efficiency was calculated by the percentage of the ratio discharge to charge capacities, according to previous reported study [58]. Clearly, palladium and Pt_80_Pd_20_ show the highest values of charge and discharge capacities, with a Faradaic efficiency of about 78 % for the bimetallic system. In presence of Nafion the highest charge capacity is still shown by the bimetallic system with a Faradaic efficiency of about 72%. The highest efficiency is presented by the platinum system with a value of 86.6%. This finding is related to the platinum NPs dispersion operated by the Nafion, according to the morphology reported in Figure 2d. However, XPS analyses revealed that the platinum surface concentration in Pt-GP is 18 times higher than that of palladium in Pd-GP and 9 times higher than that of the sum of palladium and platinum in the bimetallic system (vide Table 2). Then, the highest charge-discharge capacity is presented by the systems containing palladium. Moreover, the high ability to reduce the H^+^ ion and thus the charge capacity of the bimetallic system, according to the XPS data, is consistent with the interaction between the two metals that results in an electron donation from platinum to palladium. Table 4 reports the performances of hydrogen evolution and adsorption of the nanoelectrode arrays, described in this work, in comparison with some platinum and palladium nanoparticles-decorated carbon nanomaterials reported in literature. Since the literature data concerning the hydrogen electro-sorption and storage are often inhomogeneous, we have reported only those parameters that can be compared. In particular, Table 4 compares some nanosystems obtained by conventional chemical reduction with those obtained by PLAL. Furthermore, the fifth column of the Table 4 shows the absorption data at the pressure of 1 bar and ambient temperature, obtained by gaseous phase chemical reaction or by electro-sorption reaction, respectively. The comparison of the data shows that our systems are competitive with respect to the other state of the art materials, also taking into account the ease of preparation method proposed and the low environmental impact presented by the synthesis process, which does not involve the formation of harmful chemical synthesis waste. Furthermore, our data, according to the literature, confirm the superior performances of palladium-based nanosystems compared to platinum-based ones towards the electro-sorption reaction of H^+^.

## 4. Conclusions

In this work we propose new nanoelectrode arrays for the electro-sorption of hydrogen in an alkaline electrolyte. The nanoelectrodes consist of hybrid metal NPs-decorated graphene paper. A comparative study between naked and surrounded by a thin layer of perfluro-sulfone ionomer metal NPs has been described. Graphene paper is a lightweight carbon-based material having a high defect density that facilitates the allocation of hydrogen both as H_2_ or H by spillover mechanism. Metal NPs increase the Faradaic efficiency of the GP electrodes. The studied systems are suitable both for the production via water splitting as well as for the storage of hydrogen. The metal NPs act as catalysts for the evolution of hydrogen through the reduction of H^+^ and the GP acts as a lightweight and efficient hydrogen storage system. Significant spillover mechanism was clearly identified by the cyclic voltammograms. We described in this study that palladium surface segregation observed in the Pt_80_Pd_20_ and the electron donation from platinum to palladium increases the electro-catalytic efficiency of this system both in the charge as well as in the discharge processes with respect to the single metals alone. The Faradaic efficiency is also increased. The most significant effects of the ionomer consist mainly in the promotion of the NPs dispersion onto the surface of GP and in the increase of the ionic activity of H^+^ close to the NPs surface. Taking into account the different amount of platinum, palladium and Pt_80_Pd_20_ NPs in the studies electrodes, the latter two systems show the highest charge-discharge capacities. Furthermore, the ionomer acts as H^+^ selective membrane producing an evident increase of the charge-discharge capacities of the hybrid electrodes in alkaline electrolyte. In future work a thinner GP support will be taken into account to increase the energy density of the proposed nanoelectrode arrays.

## Figures and Tables

**Table 1:** Position (Binding Energy/eV) of the peak components used in the deconvolution of the spectra reported in Figure 5 and Figure 6.

Sample	C 1s				O 1s		Pt 4f_7/2,5/2_			Pd 3d_5/2,3/2_	
	C *sp^2^*	C-OH	C=O	O-C=O	C-O_x_H	H_2_O	Pt^0^	Pt(II)	Pt (IV)	Pd^0^	Pd(II)
Pd-GP	284.6	285.8	287.1	288.4	531.5	535.5	-	-	-	335.5/340.8	337.5/342.8
Pt-GP	284.6	286.0	287.1	288.3	531.1	533.1	71.3/74.6	72.6/76.0	75.2/78.5	-	-
Pt_80_Pd_20_GP	284.6	285.7	286.9	288.5	531.8	534.0	71.3/74.6	72.5/75.9	75.0/78.3	335.5/340.8	336.8/342.1

**Table 2:** Composition of the surfaces of the NPs-GP systems obtained by XPS (atomic concentration %).

Sample	C 1s (tot.)	O 1s (tot.)	Pt 4f_7/2,5/2_ Pt^0^+Pt(II)+Pt(IV)	Pd 3d_3/2,1/2_ Pd^0^+Pd(II)
Pd	75.5	22.9	-	1.6
Pt	49.2	20.9	29.9	-
Pt_80_Pd_20_	80.3	16.4	2.0	1.3

**Table 3:** Charge—discharge specific capacity (Ahg^−1^) and Faradaic efficiency of the NPs-GP systems. Conditions: KOH 1 M; current ± 100 μA.

System	Suspension Medium of NPs	Charge (−0.3 V)	Discharge (+0.25 V)	Faradaic Efficiency %
GP alone	-	4.65	2.77	59.6
Pt-GP	water	4.64	2.98	64.2
Pd-GP	water	5.60	3.70	66.1
Pt_80_Pd_20_-GP	water	5.16	4.02	77.9
Pt-GP	water-0.25 wt.% nafion	5.60	4.85	86.6
Pd-GP	water-0.25 wt.% nafion	5.77	2.76	47.8
Pt_80_Pd_20_-GP	water-0.25 wt.% nafion	6.21	4.50	72.4

**Table 4:** Performances of hydrogen evolution and adsorption of the systems described in this work in comparison with some platinum and palladium nanoparticles-decorated carbon nanomaterials reported in literature.

System	Electrode	Metal NPs Production Method	Electrolyte (ElectroChemical Method) or Gaseous Phase Reaction of H_2_ Adsorption	Hydrogen Storage/Evolution (Wt.%)	Faradaic Efficiency (%)	Reference
Pd NPs/nafion	GCE	Wet/NaBH_4_	H_2_SO_4_	0.003	83.1	[52]
Pd-rGO/nafion	GCE	Wet/NaBH_4_	H_2_SO_4_	0.14	85	[52]
Pd/B-rGO/nafion	GCE	Wet/NaBH_4_	H_2_SO_4_	0.35	95	[52]
Pt- (GO)/HKUST-1	-	Wet/Ethylene glycol	Gaseous phase reaction	1.6	-	[12]
Pt Covalent triazine framework(CTF-N)	Fluorine doped tin oxide (FTO)	Wet/NaBH_4_	Trietanolamine ^(1)^	0.2	-	[16]
Pd Covalent triazine framework(CTF-N)	Fluorine doped tin oxide (FTO)	Wet/NaBH_4_	Trietanolamine ^(1)^	1.05	-	[16]
Ni/rGO	-	Reduction in H_2_^(2)^	Gaseous phase reaction	0.007	-	[60]
Ni/Pd/rGO,	-	Reduction in H_2_^(2)^	Gaseous phase reaction	0.13	-	[60]
Ni/Ag/Pd/rGO	-	Reduction in H_2_^(2)^	Gaseous phase reaction	0.055	-	[60]
Pd/graphene	-	Reduction in H_2_^(2)^	Gaseous phase reaction	8.67 ^(3)^	-	[4]
Pd/MWCNT	-	PLAL	Gaseous phase reaction	1.2	-	[61]
Pt-GP	GP	PLAL	KOH	1	64.2	This work
Pd-GP	GP	PLAL	KOH	6	66.1	This work
Pt_80_Pd_20_-GP	GP	PLAL	KOH	6	77.9	This work
Pt-GP/nafion	GP	PLAL	KOH	1	86.6	This work
Pd-GP/nafion	GP	PLAL	KOH	6	47.8	This work
Pt_80_Pd_20_-GP/nafion	GP	PLAL	KOH	6	72.4	This work

^(1)^ Photoelectrochemical method; ^(2)^ reduction at 300 °C, H_2_ atmosphere; ^(3)^ value referred to a pressure of 60 bar, otherwise unspecified values were measured at 1 bar.

## Data Availability

Not applicable.
